# MGATAF: multi-channel graph attention network with adaptive fusion for cancer-drug response prediction

**DOI:** 10.1186/s12859-024-05987-0

**Published:** 2025-01-17

**Authors:** Dhekra Saeed, Huanlai Xing, Barakat AlBadani, Li Feng, Raeed Al-Sabri, Monir Abdullah, Amir Rehman

**Affiliations:** 1 School of Computing and Artificial Intelligence, Southwest Jiaotong University, Chengdu, 611756 Sichuan China; 2 School of Computer Science and Engineering, Central South University, Changsha, 410083 Hunan China; 3 Faculty of Computer Sciences and Information Systems, Thamar University, Dhamar, 87246 Yemen; 4 College of Computing and Information Technology, University of Bisha, Bisha, 67714 Saudi Arabia

**Keywords:** Graph neural network, Drug response prediction, Precision medicine, Bioinformatics

## Abstract

**Background:**

Drug response prediction is critical in precision medicine to determine the most effective and safe treatments for individual patients. Traditional prediction methods relying on demographic and genetic data often fall short in accuracy and robustness. Recent graph-based models, while promising, frequently neglect the critical role of atomic interactions and fail to integrate drug fingerprints with SMILES for comprehensive molecular graph construction.

**Results:**

We introduce multimodal multi-channel graph attention network with adaptive fusion (MGATAF), a framework designed to enhance drug response predictions by capturing both local and global interactions among graph nodes. MGATAF improves drug representation by integrating SMILES and fingerprints, resulting in more precise predictions of drug effects. The methodology involves constructing multimodal molecular graphs, employing multi-channel graph attention networks to capture diverse interactions, and using adaptive fusion to integrate these interactions at multiple abstraction levels. Empirical results demonstrate MGATAF’s superior performance compared to traditional and other graph-based techniques. For example, on the GDSC dataset, MGATAF achieved a 5.12% improvement in the Pearson correlation coefficient (PCC), reaching 0.9312 with an RMSE of 0.0225. Similarly, in new cell-line tests, MGATAF outperformed baselines with a PCC of 0.8536 and an RMSE of 0.0321 on the GDSC dataset, and a PCC of 0.7364 with an RMSE of 0.0531 on the CCLE dataset.

**Conclusions:**

MGATAF significantly advances drug response prediction by effectively integrating multiple molecular data types and capturing complex interactions. This framework enhances prediction accuracy and offers a robust tool for personalized medicine, potentially leading to more effective and safer treatments for patients. Future research can expand on this work by exploring additional data modalities and refining the adaptive fusion mechanisms.

## Introduction

Cancer remains one of the most significant global health challenges, affecting millions of individuals worldwide and contributing to a substantial number of deaths annually. Effective treatment strategies are crucial for improving patient outcomes, yet predicting how a particular treatment will affect a patient’s tumor response is complex. Precision medicine aims to address this challenge by tailoring treatments to individual patients, but early prediction methods often fall short due to their reliance on limited data sets, such as genetic information or protein expression levels, which do not fully capture the intricate biology of cancer [1–3]. Over the past few years, the growing accessibility of molecular data obtained from individuals affected by cancer has instigated the advancement of various extensive drug response initiatives. These endeavors, namely the GDSC [4] and CCLE [5], aim to incorporate a broad spectrum of data sources to augment the precision of prognosticating drug responses. To this end, molecular profiling has emerged as a primary technique in determining the efficacy of cancer treatments [6]. It involves a comprehensive analysis of the genetic and molecular characteristics of a patient’s tumor to gain a more profound understanding of its underlying biology. By utilizing advanced computational methods and combining multiple sources of molecular data, it is possible to derive a holistic view of the tumor and predict how it will respond to different therapies [7]. Through the integration of various data sources, researchers can overcome one of the significant limitations of early prediction methods, which rely on a single type of data, leading to inadequate insights into the underlying biology of cancer. With a more comprehensive understanding of the molecular profile of a patient’s cancer, doctors can determine the most effective treatment regimen, which is essential for precision medicine. The ability to predict drug response accurately can significantly impact the lives of cancer patients, making large-scale drug response projects [4, 5, 8] critical in advancing cancer research and treatment.

In this context, several machine learning algorithms have been employed, including linear regression [9, 10], decision trees [11, 12], random forests [13], support vector machines (SVMs) [14, 15], and neural networks [16–21]. However, these traditional machine learning algorithms suffer from various limitations that impede their effectiveness [22, 23]. For instance, the random forest algorithm’s main limitations include over-fitting and the limited information derived from genomic data alone. Likewise, the SVM algorithm’s main limitations involve being sensitive to the selection of parameters, the constraints of binary classification, and the quality and complexity of the input data. Despite the algorithm used, a common challenge in using machine learning for drug response prediction (DRP) is the complexity of the data and the intricate relationships between features and drug response, often resulting in overfitting. Unlike simple algorithms like linear regression or decision trees, complex algorithms like neural networks are challenging to interpret, which makes it difficult to understand the underlying mechanisms that drive the predictions.

To overcome the limitations of traditional machine learning in predicting cancer drug response, Convolutional Neural Networks (CNNs) [3, 24, 25] have emerged as a promising alternative. Convolutional Neural networks (CNNs) are engineered to automatically capture and assimilate salient features from input data, effectively manage complex and high-dimensional data, possess the ability to withstand noise and variability, and incorporate the spatial relationships that exist between features. As a result, CNNs are particularly adept at analyzing cancer genomics data and hold promise for enhancing the precision and accuracy of DRP. Nevertheless, the performance of CNN models is partially contingent upon the structure of the molecular data, thereby producing a level of prediction accuracy that is constrained. This limitation stems from the fact that CNNs represent drugs as strings, which does not align with the natural structure of drugs. Recently, graph neural networks (GNNs) have demonstrated significant potential in cancer drug response prediction [26–32], showcasing encouraging outcomes. GNNs are specifically designed to handle data organized in a graph structure, where the nodes represent unique entities and the edges depict the connections or associations among them. In the domain of cancer drug response, drug composition can be represented as a graphical structure where atoms are nodes and the bonds between them are edges. Employing GNNs in this context offers significant benefits, as they can effectively integrate graph-based information into the model. This integration enables GNNs to make more precise predictions, enhancing the accuracy of the overall analysis. Additionally, GNNs can handle sparse and noisy data, making them well-suited for molecular data. Existing graph neural network (GNN) models for DRP encounter several limitations that hinder their performance. Firstly, many GNNs have limited model capacity, often being shallow networks that primarily capture local graph structures. This deficiency restricts their ability to handle intricate relationships among nodes, potentially leading to decreased accuracy in DRP. Secondly, the prevalent reliance on a single-layer attention mechanism in most GNN models proves insufficient for capturing the nuanced interconnections within graphs. These weaknesses undermine their capability to accurately leverage complex relationships and dependencies among nodes, limiting their suitability for DRP. Furthermore, prevalent graph-based methods often overlook the significance of integrating drug fingerprints alongside drug SMILES in constructing molecular graphs. Drug fingerprints, which encapsulate structural and physicochemical properties, offer valuable insights into molecular characteristics that influence drug responses. These fingerprints encode essential information about molecular structure, such as functional groups, bond types, and spatial arrangements, providing a nuanced understanding of drug interactions at the atomic level. Despite the rich information embedded within drug fingerprints, their incorporation into graph-based models has been limited. Instead, many existing approaches rely solely on drug SMILES representations, which primarily capture molecular connectivity. SMILES strings offer a standardized representation of molecular structures but lack detailed molecular properties essential for accurate drug response predictions. Current graph-based methods often ignore drug fingerprints, leading to suboptimal predictive performance. Integrating drug fingerprints with SMILES enhances the models’ comprehensiveness and predictive power, resulting in more accurate drug efficacy predictions.

In response to these constraints, we introduce a Multimodal Multi-channel Graph Attention Network with Adaptive Fusion (MGATAF), an innovative framework for predicting cancer drug response. MGATAF stands out for its ability to effectively capture the intricate relationships between drugs and genes, as well as the dependencies between different drugs and genes. The current innovative approach is the process of leveraging multi-channel graph attention and adaptive fusion within MGATAF. Firstly, MGATAF constructs multimodal molecular graphs that incorporate both SMILES and drug fingerprints, providing a comprehensive representation of drug molecules. Then, multi-channel graph attention mechanisms are applied to capture complex interactions among graph nodes, allowing MGATAF to learn multiple orders of relationships. Finally, adaptive fusion techniques integrate these interactions at various levels of abstraction, enhancing prediction performance. By employing multi-channel graph attention with adaptive fusion, MGATAF offers superior predictive capabilities compared to existing methods. These contributions collectively position MGATAF as a promising advancement in the field of drug response prediction, offering enhanced predictive capabilities for precision medicine initiatives.

The main contributions of our proposed approach, as outlined in this study, are summarized as following:

1. **Introduction of MGATAF Framework**: MGATAF, a Multi-channel Graph Attention Network with Adaptive Fusion, is introduced to enhance predictive accuracy in drug response tasks by capturing local and global drug–cell interactions.
2. **Novel Attention and Fusion Modules**: MGATAF features a multi-channel graph attention mechanism and an adaptive fusion module, integrating diverse molecular data to produce robust drug–cell interaction representations.
3. **Superior Empirical Performance**: Experiments on GDSC and CCLE datasets show MGATAF outperforms state-of-the-art methods, achieving better Pearson correlation coefficients and root mean square errors.
4. **Linking Technical Innovation to Clinical Impact**: Ablation studies confirm the efficacy of the attention and fusion modules, contributing to more accurate predictions. These improvements have clinical relevance, offering more precise drug efficacy predictions to guide treatment decisions and streamline drug development.

## Related work

Cancer drug response prediction is crucial in precision medicine, guiding the selection of optimal therapeutic interventions. A wide range of methods has been employed, including traditional approaches, classical machine learning models, and recent advances in graph neural networks (GNNs). This section reviews these methods, with an emphasis on machine learning and GNN approaches relevant to our proposed model.

### Traditional approaches

Traditional methods, such as genomic profiling, biomarker-based approaches, and in vitro/in vivo models, have played a significant role in early drug response prediction research. Genomic profiling utilizes high-throughput technologies to identify genetic alterations in cancer cells, assuming a correlation between these alterations and drug responses [33, 34]. For example, EGFR mutations are associated with positive responses to EGFR-targeted therapies. While effective in some cases, the predictive power of these methods is limited by the quality of genetic data, high costs, and computational complexity.

Biomarker-based approaches aim to predict responses based on specific biological markers like proteins or gene expressions [35, 36]. However, their generalizability is often constrained by the cancer type and available biomarkers, reducing their broader applicability. Similarly, in vitro cell line models [37, 38], while useful for screening drug sensitivity, fail to capture the complex biological characteristics of tumors in patients, limiting their clinical relevance.

### Machine learning-based approaches

Classical machine learning models, including support vector machines (SVMs) and matrix factorization techniques, have been applied to drug response prediction with varying degrees of success. Dong et al. [39] employed SVMs with recursive feature selection to predict drug response across multiple cell lines, while Wang et al. [40] introduced a similarity-regularized matrix factorization (SRMF) method to enhance the predictive power by leveraging the similarity between cell lines and drugs. Despite their utility, these approaches often struggle to capture the full complexity of molecular interactions, leading to limited prediction accuracy.

Several computational models have been developed to predict miRNA-disease associations by integrating heterogeneous biological data and employing advanced machine-learning techniques.

Chen et al. [41] introduced the DBNMDA model, which utilizes deep-belief networks for miRNA-disease association prediction. By pre-training restricted Boltzmann machines on feature vectors constructed from all miRNA-disease pairs and fine-tuning with both positive and selected negative samples, the model effectively leverages information from both known and unknown associations. This approach reduces the impact of limited known associations on prediction accuracy and achieves superior performance, with high AUC scores in various cross-validation settings and successful case studies.

Ha et al. [42] proposed IMIPMF, employing probabilistic matrix factorization to predict miRNA-disease associations. By drawing an analogy to recommender systems, their method addresses the challenge of predicting associations for new miRNAs and diseases without prior known associations. IMIPMF demonstrates high performance with a reliable AUC value, highlighting its effectiveness despite only considering known miRNA-disease associations and miRNA expression data. Another innovative framework is NCMD, which [43] utilizes node2vec-based neural collaborative filtering for miRNA-disease association prediction. This method learns low-dimensional vector representations of miRNAs and diseases using Node2vec and combines the linear capabilities of generalized matrix factorization with the nonlinear abilities of a multilayer perceptron. Extensive experiments and case studies validate its effectiveness in discovering novel miRNA-disease associations. Ha [44] introduced MDMF, a computational framework that predicts miRNA-disease associations using matrix factorization with a disease similarity constraint. By integrating heterogeneous information and evaluating performance through global and local leave-one-out cross-validation, MDMF achieves significant improvements over previous methods. Case studies on major human cancers further demonstrate its efficiency in uncovering miRNA-disease associations and deciphering the roles of miRNAs in disease pathogenesis.

In the SMAP framework proposed by Ha [45], miRNA-disease associations are identified by applying recommendation algorithms with miRNA and disease similarity constraints. By measuring comprehensive similarity values based on miRNA functional similarity, disease semantic similarity, and Gaussian interaction profile kernel similarity, SMAP effectively integrates known associations and similarities to achieve high AUC scores in cross-validation and case studies, serving as a guide for elucidating disease pathogenesis and biomarkers. Ha [46] also presented MLMD, a metric learning-based model for predicting miRNA-disease associations. MLMD learns miRNA-disease metrics to uncover novel associations as well as miRNA-miRNA and disease-disease similarities. The model demonstrates outstanding performance compared to state-of-the-art methods, with reliable AUC scores in cross-validation frameworks and successful case studies confirming its practicality and feasibility.

Furthermore, Ha [47] extended computational approaches to lncRNA-disease associations by proposing EMFLDA, a matrix factorization method that applies lncRNA expression profiles. By effectively incorporating heterogeneous biological datasets and using expression profiles as weights, EMFLDA improves model accuracy and performance. The model outperforms previous methods in AUC scores and plays a pivotal role in extracting disease biomarkers.

### Graph neural network approaches

Recent advancements in graph neural networks (GNNs) have shown great promise in drug response prediction by effectively modeling the intricate relationships between drugs and cancer cell lines.

Several studies have applied GNNs to drug response tasks. For instance, Yang et al. [48] developed GPDRP, a multimodal framework leveraging drug molecular graphs and gene pathway activity for drug response prediction, while Wang et al. [49] proposed the XMR model, an explainable multimodal neural network for predicting drug efficacy. While attention-based GNNs [29, 50] have improved predictive accuracy by enhancing the model’s ability to learn from molecular representations, their reliance on single-layer attention mechanisms has limited their capacity to capture complex relationships across multiple layers. These models often fail to incorporate diverse neighboring orders of information within the graph structure.

To overcome this limitation, the Multi-channel Graph Attention module introduced in our work seeks to incorporate knowledge from multiple neighboring orders into the final prediction. This allows for a more comprehensive understanding of drug–cell interactions by considering both proximal and distant relationships within the molecular graph, improving prediction accuracy. Unlike AMD-GNN [51], which primarily addresses the over-smoothing issue in deep graph neural networks for tasks such as node classification, our method, MGATAF, is specifically designed for molecular graph representation. While AMD-GNN employs decoupled propagation and transformation mechanisms, MGATAF integrates both SMILES and molecular fingerprints through a multi-graph attention framework. This allows MGATAF to capture molecular interactions across multiple data types, offering a unique approach for analyzing biochemical structures. These differences reflect the distinct goals and applications of the two methods.

## Dataset and preprocessing

### Datasets

To evaluate our model, we conducted experiments using two datasets: the Genomics of Drug Sensitivity in Cancer (GDSC) [52] and the Cancer Cell Line Encyclopedia (CCLE) [5]. Dataset descriptions are presented in Tables 1 and 2. In this study, we used the GDSC and CCLE datasets independently for all analyses. Future work could explore the potential benefits of integrating these datasets to leverage complementary information.

(1) GDSC is a large-scale initiative that screens cancer drugs to assess their efficacy on numerous cancer cell lines, while also providing corresponding omics and drug response data. In our study, we use version 6.0 of the GDSC dataset, which contains the half maximal-inhibitory concentration (IC50) values for drug–cell line pairs, covering 250 drugs and 1074 cell lines. Additionally, the cancer cell lines in the GDSC dataset are described by their genetic and omics features, such as copy number variations and mutation statuses. Drugs are identified by their names and compound ID (CID), which can be used for cross-referencing with other databases. The molecular structures of the drugs were sourced from PubChem [53].

(2) The CCLE dataset [5] provides comprehensive genomic and pharmacological data for human cancer cell lines. This dataset includes experimental data such as drug targets, dosage information, log(IC50) values, and effective area measurements for drug–cell pairs involving 24 drugs and 1036 cell lines. In our study, we utilize the log(IC50) value as the primary measure of drug sensitivity.

### Preprocessing

Following the methodology described in [54], we selected only drugs with available IC50 values. For the molecular structure of the drugs, we obtained SMILES strings from PubChem and represented them as molecular graphs, where atoms form the nodes and bonds define the edges. Each atom node was described using a 78-dimensional feature vector. We removed cell lines lacking omics data, as well as drugs that had identical compound IDs (CID) in PubChem. Additionally, drugs without a corresponding PubChem ID in the GDSC database were excluded. After preprocessing, the GDSC dataset contained 172,114 drug–cell pairs derived from 223 drugs and 948 cell lines. Of the $223 \times 948 = 211{,}404$ possible drug–cell interactions, approximately 18.6% were missing. The IC50 values of each drug–cell pair were normalized to a range between 0 and 1. Similarly, the CCLE dataset, after preprocessing, contained 11,104 drug–cell pairs involving 23 drugs and 436 cell lines. For the $24 \times 436 = 10{,}464$ possible drug–cell pairs, no interaction was missing. At the input stage, drugs were represented using their canonical SMILES format, and cell lines were encoded as a binary 735-dimensional vector.

**Table 1:** Description of the GDSC and CCLE datasets

Characteristic	GDSC	CCLE
*Before Preprocessing*		
Number of Cell Lines	1074	1036
Number of Drugs	250	24
*After Preprocessing*		
Number of Cell Lines	948	436
Number of Drugs	223	23
Drug–Cell Line Pairs	172,114	10,464
Missing Pairs	18.6%	None

**Table 2:** Atom and cell-line feature descriptions

Features	Description
*Atom*	
Atom type	C, N, O, S, F, etc. (one-hot)
Degree	0–10 (one-hot)
Implicit valence	0–6 (one-hot)
Formal charge	Formal charge number (integer)
Radical electrons	Number of radical electrons (integer)
Hybridization	SP, SP2, SP3, SP3D, SP3D2 (one-hot or null)
Aromatic	Whether the atom is in an aromatic system (binary)
Hydrogens	0–4 (one-hot)
Ring	Whether the atom is in a ring (binary)
Chirality	R, S (one-hot or null)
*Cell-line*	
Gene expression	Expression levels of genes in cell lines
Copy number variation	Variations in the number of copies of a gene
Somatic mutations	Presence or absence of mutations in genes

## Method

**Fig. 1:**
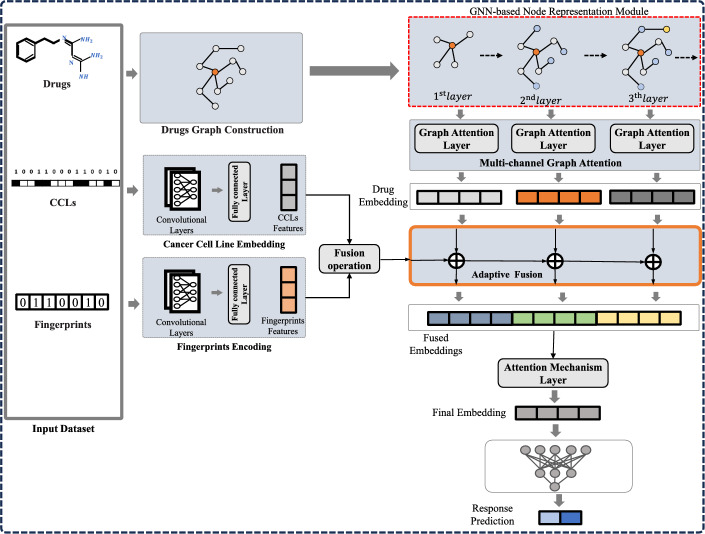
MGATAF framework

The MGATAF framework comprises three primary modules, namely the GNN-based node representation module, the multi-channel graph attention module, and the adaptive fusion module, as illustrated in Fig. 1. In this particular section, our initial focus will be on presenting an outline of the framework in its entirety, and this will be followed by a detailed description of each individual module included in it.

### Overview

In Fig. 1, the GNN-based node representation module is depicted, which accepts the drug graph and employs a GNN-based network with multiple layers to obtain the drug graph’s representation. This module retains all node embeddings from each layer. The multi-channel attention graph module begins by creating a virtual super node $c_s$, which is linked to all drug nodes. A graph neural network is then used to generate the graph representation of the super node $c_s$. Next, the hidden state of $c_s$, corresponding to the graph embedding of the node embedding layer, is updated using GRU. An attention mechanism is utilized to determine the weight of each drug’s graph embedding. The CCL and fingerprint features are generated by the cancer cell line embedding and fingerprint encoding modules, respectively. Then, an adaptive fusion module is proposed to match the drug representation and fused CCL and fingerprint representations. This module extracts the correlation from both local and global perspectives, resulting in a comprehensive representation of cancer and drug responses. This enables effective integration of the encoded drug representation with the encoded features of cancer cell lines and fingerprints. Furthermore, an attention mechanism layer is employed to identify the most significant features of the fused embeddings. The output is then generated using a fully connected layer.

### GNN-based node representation module

GNNs are an effective approach for extracting knowledge from data that is structured as graphs [55]. GNNs possess the capability to learn from graph-structured data, such as social networks, molecular structures, and other types of networks. By effectively capturing the complex relationships between nodes within a graph, GNNs can leverage this information to make predictions or provide recommendations. Given the drug graph $G_d= (A_d, X)$, where *X* is the node feature matrix and $A_d$ is the adjacency matrix of graph. A GCN function is applied to generate the output representation $Z^l_d$ of the *l*-th layer, which can be represented as follows:

1 $$\begin{aligned} Z_d^{(l)} = ReLU ({\tilde{D}}_d^{-\frac{1}{2}} {\tilde{A}}_d {\tilde{D}}_d^{-\frac{1}{2}} {\tilde{Z}}_d^{(l-1)}{\tilde{W}}_d^{(l)}), \end{aligned}$$

where ${\tilde{W}}_d^{(l)}$ is the weight matrix of *l*-th layer in GCN, the initial $Z_d^{(0)} =X$, *ReLU* indicate the Relu activation function, ${\tilde{A}}_d= {A}_d + {I}_d$, and ${\tilde{D}}_d$ means the diagonal degree matrix of ${\tilde{A}}_d$.

### Multi-channel graph attention module

This section describes the multi-channel graph attention module, which is one of the key components of our proposed framework. The Multi-channel graph attention module is designed to effectively learn representations that take into account multiple levels of information about the relationships between nodes in a graph. This module utilizes the power of graph neural networks (GNNs) in combination with a multi-channel graph attention technique to learn and extract informative representations of graphs. Specifically, a graph’s intricate relationships are captured by applying multiple layers of attention. The underlying idea is to use multiple layers of attention to capture different levels of relationships between nodes in the graph, thereby allowing us to capture and encode complex and multi-scale patterns in the graph. Each layer of attention captures different types of relationships from different layers in the network. This enables us to capture all essential dependencies for modeling complex graphs. Overall, our proposed multi-channel graph attention module aims to enhance the expressive power of our framework, enabling us to learn more informative and powerful representations of graph-structured data. By incorporating the various types of relationships captured by different layers of attention, the proposed model can acquire a more comprehensive and intricate graph representation. This is because the different layers of attention are designed to capture distinct levels and types of relationships between nodes in the graph, allowing the model to learn and incorporate more intricate patterns and dependencies among the nodes. Thus, by combining these different types of relationships, the model can learn more complex graph representations that can capture the intricacies of the data. Specifically, this module will receive the output representation of each layer, $Z^l_d= \{ z_1, z_2, \dots , z_N\}$, $z_i \in {\mathcal{R}}^D$, where *N* is the number of nodes, and *D* is the feature vector dimension. This module generates new node features for each layer received from the GNN-based node representation module and produces a new set of node features, ${Z}'= \{ z'_1, z'_2, \dots , z'_N\}$, $z'_i \in {\mathcal{R}}^{D'}$.

The attention coefficient $e_{ij}$, indicating the significance of node *j*’s features to node *i*, can be calculated as follows:

2 $$\begin{aligned} e_{ij} = \alpha (Wz_i, Wh_j), \end{aligned}$$

where $W \in {\mathbb{R}}^{F' \times F}$ is a weight matrix, and a represents a shared attention mechanism: ${\mathbb{R}}^{F'} \times {\mathbb{R}}^{F'} \rightarrow {\mathbb{R}}$. In the experiment, this mechanism is implemented as a single-layer feedforward neural network.

Using the attention mechanism, the model can assign different weights to drugs depending on their relevance to the graph embedding. This helps to capture the complex relationships between drugs and their interactions within the graph. The resulting graph embedding is a compact representation of the entire graph that can be used for prediction tasks. To ensure the coefficients are easily comparable across different nodes, the SoftMax function is used to normalize them for all choices of *j*:

3 $$\begin{aligned} \alpha _{ij} = softmax_{j} (e_{ij}) = \frac{exp(e_{ij})}{\sum _{k\in N{i}} exp(e_{ik})} \end{aligned}$$

where $N_i$ is the set of node *i*’s neighbors in the graph. Additionally, a is parameterized by a weight vector $\varphi \in {\mathbb{R}}^{2D'}$, with the nonlinearity Leaky ReLU serving as the activation function.

4 $$\begin{aligned} \alpha _{ij}= \frac{exp( LeakyReLU (\varphi ^{T}[Wz_i\parallel Wz_j]))}{\sum _{k\in N_i}exp( LeakyReLU (\varphi ^{T}[Wz_i\parallel Wz_k]))} \end{aligned}$$

where $.^T$ is the transposition operation. Then, the output of node representation *i* based on the multi-head attention mechanism is as follows:

5 $$\begin{aligned} z'_i = \parallel _{k=1}^{K}\sigma \left( \sum _{j \in N_i} \alpha _{ij}^k W^k z_i\right) \end{aligned}$$

where *K* and $\sigma$ represent the number of attention mechanism heads and the nonlinear function, respectively. Additionally, $\sigma ^{k}_{ij}$ are the normalized attention coefficients computed by the *k*-th attention mechanism ($\sigma _{k}$), and $W^k$is the corresponding weight matrix for the input linear transformation. In the final layer of the network, an average multi-head attention mechanism is applied as follows.

6 $$\begin{aligned} h'_i = \sigma \left( \frac{1}{K} \sum _{k=1}^{K} \sum _{j \in N_i} \alpha _{ij}^k W^{k} z_j\right) \end{aligned}$$

where $\sigma$ is the activation function.

Finally, the multi-channel graph attention module is the graph embedding for multi-level representing the embedding of multiple layers, as follows:

7 $$\begin{aligned} {[}h_1^l, h_2^l, \dots , h_N^l] = h_i^l = \sigma \left( \frac{1}{K} \sum _{k=1}{K} \sum _{j \in N_i} \alpha _{ij}^k W^{k} z_j\right) \end{aligned}$$

where *l* is the layer number.

### Cancer cell line embedding and fingerprints encoding

For a given cancer cell line *C*, let’s denote *C* as a matrix with dimensions (*N*, *F*), where *N* is the number of features (e.g., the number of features) and *F* is the number of features per cell line. The output of a convolutional layer can be computed as follows:

8 $$\begin{aligned} C_{ij} = \sigma \left( \sum _{k=1}^{F} \sum _{l=1}^{w} C_{(i+l-1), k} \cdot W_{j, k, l} + b_j \right) \end{aligned}$$

$C_{ij}$ is the output feature map at position *i* and channel *j*, $\sigma$ is the activation function (e.g., ReLU), *w* is the size of the convolution filter (kernel), $W_{j,k,l}$ is the weight of the *j*-th filter at feature *k* and filter position *l*, and $b_j$ is the bias term for the *j*-th filter.

After flattening the output of the convolutional layer, the output of the fully connected layer can be expressed as follows:

9 $$\begin{aligned} C_i = \sigma \left( \sum _{j=1}^{M} C_{j} \cdot W_{ij} + b_i \right) \end{aligned}$$

$C_i$ is the output of the *i*-th neuron in the fully connected layer, $\sigma$ is the activation function (e.g., ReLU, Sigmoid), *M* is the number of inputs to the fully connected layer (flattened feature maps from the convolutional layer), $C_j$ is the *j*-th input to the fully connected layer, $W_{ij}$ is the weight connecting the *j*-th input to the *i*-th neuron, and $b_i$ is the bias term for the *i*-th neuron.

For a given fingerprint *F*, let’s denote *F* as a matrix with dimensions (*M*, *K*), where *M* is the number of features (e.g., the number of features) and *K* is the number of features per cell line. The output of a convolutional layer can be computed as follows:

10 $$\begin{aligned} F_{ij} = \sigma \left( \sum _{k=1}^{K} \sum _{l=1}^{w} C_{(i+l-1), k} \cdot W_{j, k, l} + b_j \right) \end{aligned}$$

$F_{ij}$ is the output feature map at position *i* and channel *j*, $\sigma$ is the activation function (e.g., ReLU), *w* is the size of the convolution filter (kernel), $W_{j,k,l}$ is the weight of the *j*-th filter at feature *k* and filter position *l*, and $b_j$ is the bias term for the *j*-th filter.

After flattening the output of the convolutional layer, the output of the fully connected layer can be expressed as follows:

11 $$\begin{aligned} F_i = \sigma \left( \sum _{j=1}^{M} C_{j} \cdot W_{ij} + b_i \right) \end{aligned}$$

$F_i$ is the output of the *i*-th neuron in the fully connected layer, $\sigma$ is the activation function (e.g., ReLU, Sigmoid), *M* is the number of inputs to the fully connected layer (flattened feature maps from the convolutional layer), $F_j$ is the *j*-th input to the fully connected layer, $W_{ij}$ is the weight connecting the *j*-th input to the *i*-th neuron, and $b_i$ is the bias term for the *i*-th neuron.

Then, the cancer cell lines embedding and fingerprints embedding are concatenated as follows:

12 $$\begin{aligned} X = concat[C, F] \end{aligned}$$

where *C* is the cancer cell embeddings and *F* is the fingerprints embeddings.

### Adaptive fusion module

**Fig. 2:**
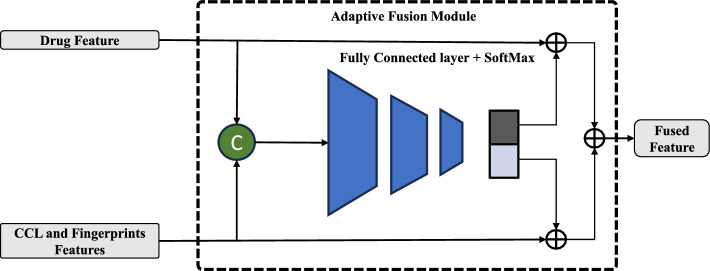
Adaptive fusion module structure

Adaptive fusion is a technique in neural networks that involves the merging of several data sources into a single output. The objective of this approach is to improve the precision and reliability of the neural network by integrating diverse data sources, such as drug representation and cancer cell line representation, thereby enhancing its accuracy and stability. By doing so, the model can extract complementary information from different sources and produce more comprehensive and reliable predictions. The combination process involves dynamically weighting the different data sources based on their relative importance and relevance to the task at hand. The adaptive nature of the technique ensures that the fusion process is tailored to the specific input data and task, making it more effective and efficient than fixed fusion methods. As shown in Fig. 2, the proposed adaptive fusion module integrates multiple input representations from both representations into a single output. This process can be done using a weighted sum operation. The weights assigned to each input are adjusted based on the performance of the network on a given cancer and drug response prediction task. This allows the network to adapt to changing conditions and improve its accuracy over time. Adaptive fusion can also be used to reduce overfitting by combining multiple sources of information into one output.

In the last layer, the study combines the representations of the drug and cancer cell line to capture their overall relationship. This comprehensive method allows us to create a powerful representation by capturing the correlation between them. To accomplish this, the study employs an adaptive fusion module that integrates the two-view features using a control gate weight. This weight is determined by the underlying temporal properties of the features, which are processed through a fully connected layer followed by a Sigmoid function. The output of the Sigmoid function represents the control gate weight, which is then used to modify the two-view features. This modification ensures that the most informative features are emphasized while irrelevant features are suppressed. By employing this technique, the study aims to enhance the accuracy and robustness of the neural network by effectively integrating multiple sources of information. The two modified characteristics are then combined. The AF module is formulated as follows:

13 $$\begin{aligned} g_{out}= & \sigma (W_g (concat[X,H]) + b_g), \end{aligned}$$

14 $$\begin{aligned} H= & (h \bigotimes g_{out_1} \bigoplus X \bigotimes g_{out_2}), \end{aligned}$$

where *H* is the fingerprint representations, *contact* represents concatenation, $W_g$ is the FC layer’s trainable parameter, and $b_g$ is the bias term. $\sigma$ is the SoftMax function, *h* represents the learned embeddings for the input data, while $g_{out1}$ and $g_{out2}$ are the gating mechanisms responsible for modulating the importance of the fused features in the adaptive fusion module.

Finally, an attention mechanism is applied and fully connected layer as follows:

15 $$\begin{aligned} e_{ij} = \text {LeakyReLU} \left( {\textbf{a}}^T \left[ {\textbf{W}} {\textbf{h}}_i \Vert {\textbf{W}} {\textbf{h}}_j \right] \right) \end{aligned}$$

where $e_{ij}$ represents the attention score between node *i* and node *j*, ${\textbf{a}}$ is the weight vector, ${\textbf{W}}$ is the weight matrix, ${\textbf{h}}_i$ and ${\textbf{h}}_j$ are the feature vectors of nodes *i* and *j*, and $\Vert$ denotes concatenation. Normalize the attention scores using the softmax function:

16 $$\begin{aligned} \alpha _{ij} = \frac{\exp (e_{ij})}{\sum _{k \in {\mathcal{N}}_i} \exp (e_{ik})} \end{aligned}$$

where $\alpha _{ij}$ is the normalized attention coefficient, and ${\mathcal{N}}_i$ denotes the neighbors of node *i*.

Then, Compute the final output embedding:

17 $$\begin{aligned} {\textbf{h}}_i' = \sigma \left( \sum _{j \in {\mathcal{N}}_i} \alpha _{ij} {\textbf{W}} {\textbf{h}}_j \right) \end{aligned}$$

where ${\textbf{h}}_i'$ is the updated feature vector for node *i*, and $\sigma$ is an activation function, such as ReLU.

The fully connected layer for the embedding $H'$ can be described as follows: Flatten the output embedding $H'$:

18 $$\begin{aligned} {\textbf{h}}' = \text {Flatten}(H') \end{aligned}$$

Then, apply the fully connected layer:

19 $$\begin{aligned} O_i = \sigma \left( \sum _{j=1}^{M} {\textbf{h}}_j' W_{ij} + b_i \right) \end{aligned}$$

where $O_i$ is the output of the *i*-th neuron in the fully connected layer, $\sigma$ is the activation function (e.g., ReLU, Sigmoid), *M* is the number of inputs to the fully connected layer, ${\textbf{h}}_j'$ is the *j*-th input to the fully connected layer, $W_{ij}$ is the weight connecting the *j*-th input to the *i*-th neuron, $b_i$ is the bias term for the *i*-th neuron.

## Experiments

### Baselines

The study assesses the effectiveness of MGATAF compared to various established models as baselines,such as tCNNS [56] and variants of popular graph neural network-based models, including GCN [57], GIN [58], GAT [59], and SuperGAT [60]. The tCNNS model employs SMILES strings to represent drugs and utilizes a convolutional layer to extract features of drugs. Additionally, it employs another convolutional layer to extract features of cancer cell lines from genetic attribute vectors. Ultimately, a fully connected layer is employed to predict the response of the drug–cell interaction.

On the other hand, the GCN, GIN, GAT, and SuperGAT models adopt a unique approach by representing drugs as graphs and cancer cell lines as one-hot vectors. These models employ graph convolutional layers to extract essential characteristics from both drugs and cancer cell lines. Subsequently, the drug and cancer cell line features are combined, and the models predict the IC50 value.

The study compares the performance of these models against MGATAF, which is a proposed approach that combines the drug and cancer cell line representations at various levels using the multi-channel graph attention module and adaptive fusion module. This allows MGATAF to capture the cross-correlation between the drug and cancer cell line representations and provide an efficient final representation for predicting the IC50 value.

### Experimental settings

To facilitate re-implementation of our MGATAF model, we provide detailed information on the parameters for each module. The MGATAF framework is composed of three primary components:

1. A **GNN-based node representation module** that employs a multi-layer Graph Convolutional Network (GCN) with ReLU activation functions, retaining node embeddings from each layer; weights are initialized using Xavier initialization, and an L2 regularization term with a weight decay of $5 \times 10 ^{-4}$ is applied.
2. A **multi-channel graph attention module** that uses multi-head attention mechanisms with 8 heads in the first layer and 1 head in the second layer, leveraging LeakyReLU activation with a negative slope of 0.2; attention coefficients are computed using shared weight matrices $W \in {\mathbb{R}}^{F' \times F}$ and attention vectors $\varphi \in {\mathbb{R}}^{2D'}$.
3. An **adaptive fusion module** that concatenates drug representations with cancer cell line and fingerprint embeddings (obtained via convolutional and fully connected layers with ReLU activations), applies a gating mechanism using a Sigmoid function to adaptively weight features and incorporates an attention mechanism followed by a fully connected layer to produce the final output.

The learning parameters are adjusted to maximize accuracy on the validation samples, with optimal values carefully selected. The entire model is trained using the Adam optimizer with a learning rate of 0.0005, a dropout rate of 0.3 applied to prevent overfitting, and early stopping if validation loss does not decrease for 50 consecutive epochs, over a maximum of 300 epochs. Training and experimentation are conducted using the NVIDIA GeForce GTX 1080 Ti graphics card. In comparison, the baseline models adhere to the same parameter settings. The experimental results are presented, with the best outcomes highlighted for clarity.

### Evalution metrics

To assess the performance of our model, we employ two metrics: the Pearson correlation coefficient (PCC) and root mean square error (RMSE). The PCC is a widely employed statistical measure that gauges the strength of the linear association between two variables. In particular, a PCC value of −1 denotes a flawless negative correlation, 0 represents no correlation, and 1 signifies a perfect positive correlation. The PCC is calculated using the following equation:

$$\begin{aligned} PCC= \frac{\sum _{i=1}^{n}(x_i-{\bar{x}})(y_i-{\bar{y}})}{\sqrt{\sum _{i=1}^{n}(x_i-{\bar{x}})^2\sum _{i=1}^{n}(y_i-{\bar{y}})^2}} \end{aligned}$$

where *x* and *Y* represent the sets of true ln(IC50) and predicted ln(IC50), respectively. *n* is the number of data points, $x_i$ and $y_i$ are the true ln(IC50) and predicted ln(IC50) of the $i^{th}$ data point, respectively, and ${\bar{o}}$ and ${\bar{y}}$ are the means of *x* and *Y*, respectively.

The RMSE is another measure of error used to evaluate the accuracy of a model in predicting quantitative data. It is calculated as follows:

$$\begin{aligned} RMSE= \sqrt{\frac{1}{n}\sum _{i=1}^{n}{(x_i-y_i)^2}} \end{aligned}$$

Here, $x_i$ and $y_i$ represent the true ln(IC50) and predicted ln(IC50) of the $i^{th}$ data point, respectively, and *n* is the number of data points in the sample.

### Experimental results

The study conducted an assessment and comparison of MGATAF’s overall effectiveness with several baseline models, specifically tCNNS and different DRP models. The study employed two metrics, PCC and RMSE, to evaluate the performance of these models. The metrics were calculated using identical benchmark datasets and settings for all approaches. In Tables 3 and 4, the study presents the outcomes of our experiments in relation to the baseline models, with the best results being emphasized in bold to facilitate easy comparison.

*Mixed Test*: In this experiment, the study assesses the performance of MGATAF using all available drugs and cell lines in the training phase, meaning that all drugs and cell lines have been seen at least once during training. This study only consider the 172114 drug–cell pairs for which response data is provided by GDSC. The data is shuffled and split into 80% as a training set, 10% as a validation set, and 10% as a test set. Table 3 shows that graph neural network-based models outperform the convolutional network-based model in both PCC and RMSE. Moreover, our approach achieves the best performance compared to all baseline models for both PCC and RMSE.

**Table 3:** Performance comparison on the GDSC and CCLE datasets in the mixed test experiment

Method	GDSC dataset		CCLE dataset	
	PCC	RMSE	PCC	RMSE
tCNNS [56]	0.8890	0.0312	0.7483	0.0612
GCN [57]	0.9118	0.0273	0.7828	0.0508
GIN [58]	0.9264	0.0252	0.7556	0.0542
GAT [59]	0.9065	0.0289	0.7741	0.0520
SuperGAT [60]	0.8800	0.0333	0.7750	0.0519
**MGATAF**	** 0.9312**	**0**.**0225**	**0.7859**	**0**.**0503**

Bold: our method.

*New Cell Line Test Experiment:* In the tests for new cells, the drugs and cells are separated into the training, validation, and test datasets instead of the interaction pairs. This simulates the scenario where new cell lines are introduced and need to be predicted. The results for these tests are shown in Table 4. The performance of all models was not as good as in the mixed experiment, indicating that it is more challenging to predict cancer drug responses for new cell lines. However, MGATAF outperforms all baseline methods in terms of both PCC and RMSE in this experiment, demonstrating its effectiveness in predicting responses for unseen cells.

**Table 4:** Performance comparison on the GDSC and CCLE datasets in the new cell-line test experiment

Method	GDSC dataset		CCLE dataset	
	PCC	RMSE	PCC	RMSE
tCNNS [56]	0.3490	0.0576	0.3469	0.0692
GCN [57]	0.8399	0.0363	0.7279	0.0563
GIN [58]	0.8460	0.0358	0.7252	0.0575
GAT [59]	0.8312	0.0380	0.7078	0.0580
SuperGAT [60]	0.8289	0.0378	0.7027	0.0585
**MGATAF**	**0.8536**	**0**.**0321**	**0**.**7364**	**0**.**0531**

Bold: our method.

We have conducted statistical significance tests to strengthen the comparison between our MGATAF model and the baseline methods. Specifically, we trained and evaluated each model five times using different random seeds to account for variability due to initialization and data shuffling and applied paired two-tailed t-tests to compare the performance of MGATAF against each baseline method on both the GDSC and CCLE datasets for the Pearson Correlation Coefficient (PCC) and Root Mean Square Error (RMSE) metrics. The results indicate that in the mixed test experiment, the improvements in PCC and reductions in RMSE achieved by MGATAF overall baseline models were statistically significant, with p-values less than 0.01 on the GDSC dataset and less than 0.05 on the CCLE dataset; similarly, in the new cell-line test experiment, MGATAF’s performance gains over the baseline methods were statistically significant, with p-values less than 0.05 for both PCC and RMSE metrics on both datasets. These statistical tests confirm that the superior performance of MGATAF is not due to random chance but is statistically significant, reinforcing the effectiveness of our proposed model in comparison to existing methods.

### Discussion

In our study, we developed the MGATAF model to predict drug responses in cancer cell lines by integrating drug representations, cancer cell line embeddings, and fingerprint features. The superior performance of MGATAF, as evidenced by the highest Pearson Correlation Coefficient (PCC) and lowest Root Mean Square Error (RMSE) in both the mixed test and new cell line test experiments (Tables 3 and 4), has significant biological implications. We can summarize the biological significance of the MGATAF model as follows:

1. **Enhanced Drug Response Prediction:** The high PCC values indicate that MGATAF can accurately predict the sensitivity of cancer cell lines to various drugs. This suggests that our model effectively captures the complex biological interactions between drug compounds and cancer cell genotypes. Accurate predictions can aid in selecting the most effective drugs for specific cancer types, thereby advancing precision medicine.
2. **Generalization to New Cell Lines:** In the new cell line test experiment, MGATAF outperforms all baseline models, demonstrating its robustness and generalization capability to unseen cell lines. This is biologically significant as it indicates the model’s potential to predict drug responses in newly discovered or less-characterized cancer cell lines, facilitating the exploration of treatment options for rare or resistant cancers.
3. **Interpretation of Molecular Mechanisms:** The multi-channel graph attention mechanism in MGATAF allows for the identification of important molecular substructures within drug compounds that contribute to their efficacy. By assigning attention weights to different parts of the drug graphs, the model highlights which molecular features are most influential, providing insights into the mechanisms of action at a molecular level.
4. **Integration of Multi-Omic Data:** The adaptive fusion module combines drug representations with cancer cell line genomic data and fingerprint features. This integrative approach reflects the multifactorial nature of drug responses, considering both the genetic makeup of the cancer cells and the chemical properties of the drugs. Such integration is crucial for understanding the complex biological pathways involved in drug sensitivity and resistance.
5. **Potential for Drug Repositioning:** The ability of MGATAF to predict responses across a wide range of drugs suggests its utility in drug repositioning efforts. By identifying unexpected sensitivities, the model can propose existing drugs as candidates for new therapeutic applications, accelerating the development of effective cancer treatments.
6. **Facilitating Biomarker Discovery:** The attention mechanisms and feature importance scores generated by MGATAF can help identify key genetic markers and molecular features associated with drug responses. This can guide experimental studies aiming to validate potential biomarkers for prognosis or as targets for new drugs.

## Ablation study

This section conducted three ablation studies to investigate the effects of multi-channel attention, adaptive fusion, and the number of layers on the overall performance of the model.

### Effect of multi-channel attention

This section presents an ablation study conducted to investigate the effect of multi-channel attention on the final representation of the model and its impact on the model’s performance. Multi-channel attention is a mechanism used in deep learning to selectively weight the importance of different channels in a multi-channel input. This study focuses on the impact of multi-channel attention on the performance of the model.

To conduct the experiment, the model was trained both with and without multi-channel attention, namely MGATAF-MA, and the final representation from the last layer of the model was used for the analysis. The representation of each layer was not kept to isolate the effect of multi-channel attention on the final representation of the model.

The results of the experiment shown in Tables 3 and 4 demonstrate that the inclusion of multi-channel attention had a positive effect on the performance of the model. This indicates that the use of multi-channel attention improves the final representation of the model and leads to better performance on the task.

It is important to note that ablation studies are typically conducted in conjunction with other experiments to provide a more comprehensive understanding of the model’s behavior. In this case, it would be useful to evaluate the performance of the model in comparison to other attention mechanisms or without any attention mechanism to further validate the effectiveness of multi-channel attention.

In summary, the ablation study conducted on the effect of multi-channel attention demonstrated that including multi-channel attention improves the final representation of the model and leads to better performance on the task. This finding underscores the importance of incorporating attention mechanisms in deep learning models to selectively weight the importance of different channels and improve the model’s performance.

### Effect of adaptive fusion

This section is dedicated to exploring the impact of adaptive fusion, which is a technique used in deep learning to merge multiple sources of information into a single output, on the performance of a model in cancer-drug response tasks. Specifically, the study is interested in exploring the differences between the concatenation operation and adaptive fusion operation in terms of performance.

The concatenation operation involves simply concatenating the features from different modalities into a single feature vector. This method can be effective in some cases, but it has the disadvantage of not being able to weigh the importance of different modalities according to their relevance to the task. On the other hand, an adaptive fusion operation is designed to dynamically adjust the importance of different modalities based on their relevance to the task, which can lead to better performance.

To conduct this ablation study, the method first trains the model using the concatenation operation as the fusion operation, namely MGATAF-AF. The study then modifies the model to use an adaptive fusion operation and compares the performance of the two models on a test set. The study aims to determine which fusion operation is more effective in capturing the complex relationships between drugs, proteins, and genes, which are critical for accurate drug response prediction.

The results of the study shown in Tables 3 and 4 demonstrate that the adaptive fusion operation leads to improved performance compared to the concatenation operation. This suggests that the ability to dynamically adjust the importance of different modalities is an important factor in achieving high performance in our tasks. Therefore, it concludes that using an adaptive fusion operation can effectively capture the complex relationships between drugs, proteins, and genes, and improve the accuracy and robustness of the model.

**Fig. 3:**
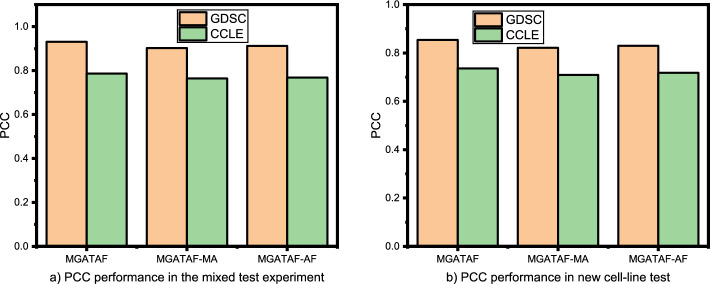
PCC performance on the effect of multi-channel attention and adaptive fusion on the GDSC and CCLE datasets in the mixed test experiment and the new cell-line test experiment

**Fig. 4:**
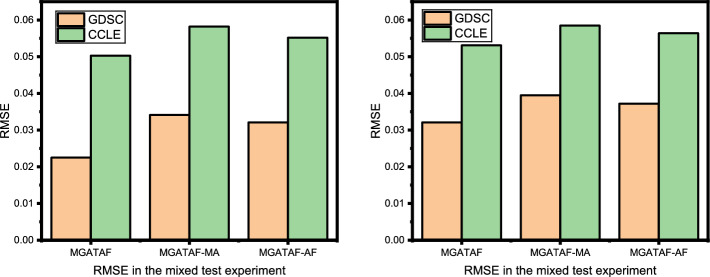
ERMS performance on the effect of multi-channel attention and adaptive fusion on the GDSC and CCLE datasets in the mixed test experiment and the new cell-line test experiment

Overall, the findings of this study highlight the importance of carefully selecting and tuning fusion operations in cancer-drug response tasks to achieve the best possible performance. By selecting the appropriate fusion operation, the method can improve the accuracy and robustness of our models, which can ultimately lead to better personalised treatments for patients as shown in Figs. 3, 4.

### Effect of number of layers

In this particular ablation study, the focus is on the effect of the number of layers on the performance of a deep learning model. The experiment involves training and evaluating models with varying numbers of layers, specifically 1, 2, 3, 4, and 5 layers.

**Fig. 5:**
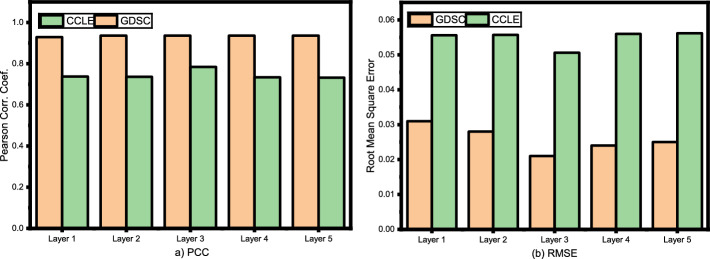
Ablation study on the number of layers

As shown in Fig. 5, as the number of layers increases, the performance of the model initially improves. This is likely due to the ability of deeper models to capture more complex patterns and relationships within the data. However, after a certain number of layers, the performance begins to decline. This phenomenon is known as the “vanishing gradient problem.” As the number of layers increases, it becomes more difficult to propagate gradients through the entire network during training, leading to slower convergence and degraded performance. The findings of this research demonstrate that the optimal number of layers for this particular problem is 3 layers. This suggests that a moderate level of depth is sufficient to capture the relevant patterns in the data, without encountering the vanishing gradient problem that can arise with deeper networks.

## Conclusion

Predicting drug response is a vital task in the field of precision medicine; however, traditional approaches have shown limited accuracy and robustness. This research presents a novel methodology known as the Multi-channel Graph Attention Network with Adaptive Fusion (MGATAF) networks, offering an innovative approach to drug response prediction. The proposed framework efficiently captures the intricate relationships among drugs, proteins, and genes, which are often overlooked by conventional methods. Additionally, it addresses the issue of disregarding the significant interactions between atoms. Our experimental findings indicate that MGATAF surpasses both traditional and graph-based methods, highlighting its potential as a robust tool for precision medicine. By enabling more precise and effective drug response predictions tailored to individual patients, our approach is poised to contribute significantly to the advancement of precision treatments, ultimately improving health outcomes. Given the promising results of this study, we anticipate that it will inspire further research aimed at enhancing patient outcomes in the field of precision medicine.

## Data Availability

The paper exclusively employs publicly accessible datasets.

## Abbreviations

MGAT: Multimodal multi-channel graph attention network
PCC: Pearson correlation coefficient
CNNs: Convolutional neural networks
GNNs: Graph neural networks
DRP: Drug response prediction
RMSE: Root-mean-square deviation
